# Adjunctive probiotics may reduce adverse events during vonoprazan-based *Helicobacter pylori* eradication: a systematic review and meta-analysis

**DOI:** 10.3389/fphar.2026.1946885

**Published:** 2026-10-19

**Authors:** Chun-Jiang Lin, Peng-Yuan Ren, Hong-Yu Chen, Ming-Qiao Zheng, Yong-Xian Jiang, Gen Li, Min Fu, Ya-Hui Guo

**Affiliations:** 1 Department of Pharmacy, Ya’an Yucheng District People’s Hospital (Second People’s Hospital of Ya’an City), Ya’an, Sichuan, China; 2 Department of Pharmacy, Sichuan Provincial Maternity and Child Healthcare Hospital and the Affiliated Women’s and Children’s Hospital of Chengdu Medical College, Chengdu, Sichuan, China

**Keywords:** adverse events, eradication therapy, *Helicobacter pylori*, meta-analysis, probiotics, tolerability, vonoprazan

## Abstract

**Background:**

Vonoprazan is pharmacologically distinct from proton pump inhibitors and is increasingly incorporated into *Helicobacter pylori* eradication regimens. Previous meta-analyses of probiotic adjuncts pooled heterogeneous acid-suppressive agents, antibiotic backbones, probiotic products, and treatment durations, limiting inference about the incremental effect of probiotics when the vonoprazan-based regimen is otherwise fixed.

**Objective:**

To determine whether adjunctive probiotics improve tolerability and eradication outcomes when randomized groups receive an otherwise identical vonoprazan-based *H. pylori* regimen.

**Methods:**

We searched eight bibliographic databases and three trial registries from inception through 15 July 2026. Eligible studies were randomized trials in adults comparing an identical vonoprazan-based regimen with or without concurrently administered probiotics. Risk ratios were pooled using restricted maximum-likelihood random-effects models with Hartung-Knapp confidence intervals. We also reported 95% prediction intervals, absolute effects, outcome-specific RoB 2 assessments, sensitivity analyses, and GRADE certainty ratings.

**Results:**

Nine studies involving 1,270 participants were included. Adjunctive probiotics reduced overall adverse events (5 studies, 611 participants; RR = 0.30, 95%CI: 0.17–0.53; P = 0.004; I^2^ = 0%; 95% prediction interval: 0.12–0.79), corresponding to 182 fewer affected participants per 1,000 treated (moderate-certainty evidence). Intention-to-treat eradication was slightly higher with probiotics (7 studies, 910 participants; RR = 1.09, 95% CI: 1.00–1.19; I^2^ = 47.8%), but the prediction interval crossed the null and sensitivity analyses were not statistically significant. Per-protocol eradication showed little or no difference (5 studies, 698 participants; RR = 1.03, 95%CI: 0.97–1.09), and good adherence was not clearly improved.

**Conclusion:**

Among Chinese adults, adjunctive probiotics probably reduce overall adverse events during vonoprazan-based *H. pylori* eradication therapy. The available evidence does not establish a reliable improvement in eradication success, and applicability to populations outside China remains uncertain.

**PROSPERO Registration:**

CRD420261450405.

## Introduction

*Helicobacter pylori* infection remains highly prevalent worldwide and is causally associated with chronic gastritis, peptic ulcer disease, gastric mucosa-associated lymphoid tissue lymphoma, and gastric cancer. Although adult prevalence has declined over recent decades, a recent global synthesis estimated that 43.9% of adults remained infected during 2015–2022 (Chen et al., 2024). International and Chinese guidelines therefore emphasize effective eradication and post-treatment confirmation of cure (Malfertheiner et al., 2022; Zhou et al., 2022; Chey et al., 2024).

Vonoprazan is pharmacologically distinct from proton pump inhibitors. As a potassium-competitive acid blocker, it provides rapid, potent, and sustained acid suppression and supports effective dual, triple, and quadruple eradication regimens. Randomized trials have demonstrated clinically useful eradication rates with vonoprazan-based dual and triple therapy in different settings (Chey et al., 2022; Hu et al., 2024). This distinct acid-suppression profile may alter both baseline regimen performance and the residual scope for benefit from adjunctive probiotics. Nevertheless, substantial antibiotic exposure remains necessary, and gastrointestinal symptoms, taste disturbance, and other treatment-related adverse events may reduce treatment acceptability and compromise completion.

Adjunctive probiotics and other defined live microbial preparations may attenuate antibiotic-associated dysbiosis, support colonization resistance, modulate mucosal responses, and reduce gastrointestinal adverse events. However, previous meta-analyses commonly combined proton pump inhibitor-, vonoprazan-, and other acid-suppression backgrounds while also pooling different probiotic strains, antibiotic regimens, treatment durations, and studies of variable quality (Yang et al., 2024). This clinical and methodological heterogeneity can obscure the incremental effect of probiotics when the underlying vonoprazan regimen is otherwise identical between randomized groups.

We therefore conducted a systematic review and pairwise meta-analysis restricted to randomized trials comparing the same vonoprazan-based *H. pylori* eradication regimen with or without adjunctive probiotics. The primary objective was to determine whether probiotics reduce the proportion of participants who experience at least one adverse event. The primary efficacy outcome was intention-to-treat eradication; per-protocol eradication, adherence, treatment discontinuation, serious adverse events, individual gastrointestinal adverse events, and exploratory microbiome outcomes were evaluated as secondary outcomes.

## Materials and methods

### Protocol, registration and reporting

This review was prospectively registered in PROSPERO (CRD420261450405), and the methods were prespecified before formal study selection and data extraction. Reporting followed the PRISMA 2020 statement (Page et al., 2021). After mapping the evidence network, we did not perform a network meta-analysis because most probiotic nodes were represented by a single study, no closed loops were available, and inconsistency could not be assessed. Quantitative synthesis was therefore limited to direct pairwise comparisons. The registered protocol is accessible through PROSPERO using the registration number.

### Eligibility criteria

Eligible studies were parallel-group randomized controlled trials that enrolled adults aged 18 years or older with confirmed *H. pylori* infection and compared a vonoprazan-based eradication regimen plus a probiotic or defined live microbial preparation with an otherwise identical regimen without probiotics or with a matching placebo. Probiotic administration had to overlap at least partly with the eradication course. Both first-line and rescue-treatment populations were eligible. Within each trial, the acid suppressant, antibiotics, bismuth, drug doses, administration frequencies, and eradication-treatment duration had to be identical between randomized groups, thereby isolating the incremental effect of probiotic supplementation.

We excluded non-randomized, clearly quasi-randomized, retrospective, single-arm, crossover, pediatric, animal, and laboratory studies. We also excluded comparisons in which randomized groups received different background eradication regimens, studies that used a potassium-competitive acid blocker other than vonoprazan, and studies in which probiotic administration began only after eradication treatment. Full articles, dissertations, conference reports, and registry reports were eligible if they provided sufficient randomized outcome data. Multiple reports from the same trial were linked and treated as a single study.

### Information sources and search strategy

PubMed, Embase, the Cochrane Central Register of Controlled Trials, Web of Science Core Collection, China National Knowledge Infrastructure, Wanfang Data, SinoMed/Chinese Biomedical Literature Database, and the VIP Chinese Science and Technology Journal Database were searched from inception through July 14–15, 2026. We also searched ClinicalTrials.gov, the Chinese Clinical Trial Registry, and the WHO International Clinical Trials Registry Platform. No restrictions were imposed on language, publication date, or publication status. Search strategies combined controlled vocabulary and free-text terms for *H. pylori*, vonoprazan or potassium-competitive acid blockers, probiotics or named live microbial products, and microbiota-related concepts. Complete platform-specific strategies are provided in Supplementary Appendix 1. Reference lists and linked registry records were checked for companion reports and ongoing studies.

### Study selection and data collection

Records were deduplicated using trial-registration identifiers, DOI, PMID, normalized title, author, publication year, journal, and pagination. Two reviewers independently screened titles and abstracts and assessed full-text reports. Disagreements were resolved through discussion, with adjudication by a third reviewer when necessary. Reports describing the same randomized population were linked under a common study identifier. Two reviewers independently extracted study design, setting, eligibility criteria, participant characteristics, randomization and masking methods, eradication regimen, probiotic product and dose, treatment duration, outcome definitions, denominators, event counts, follow-up, funding, and conflicts of interest. Discrepancies were resolved by consulting the source report and reaching consensus. When a potentially eligible registry record lacked numerical results or key eligibility details, the investigators were contacted by e-mail; one completed eligible trial remained classified as awaiting results because no response was received.

### Outcomes

The primary outcome was the proportion of randomized or treated participants who experienced at least one treatment-emergent or treatment-related adverse event during eradication therapy. We did not sum separate adverse-event episodes because an individual participant could experience more than one event. Accordingly, “overall adverse events” represented a participant-level binary outcome—whether an individual experienced at least one adverse event—rather than the cumulative number of adverse-event episodes or the sum of individual symptom categories. The primary efficacy outcome was *H. pylori* eradication in the intention-to-treat population or the closest available analysis that retained the randomized denominator. Secondary outcomes were per-protocol eradication, good adherence or treatment completion, discontinuation because of adverse events, serious adverse events, and individual gastrointestinal adverse events. Microbiome outcomes were prespecified as exploratory and summarized narratively by sampling site and time point because the reported metrics were not sufficiently comparable for quantitative synthesis. Ambiguous overall-adverse-event totals were not assumed to represent unique affected participants; such data were excluded from the primary synthesis and considered only in sensitivity analyses when extractable.

### Risk of bias and certainty of evidence

Two reviewers independently assessed outcome-specific risk of bias using the Cochrane Risk of Bias 2 tool (Higgins et al., 2024; Sterne et al., 2019). The five domains addressed the randomization process, deviations from intended interventions, missing outcome data, outcome measurement, and selection of the reported result. Overall judgments were categorized as low risk, some concerns, or high risk. Lack of blinding was considered particularly relevant to subjective adverse-event reporting, whereas objectively confirmed eradication by urea breath testing was less susceptible to measurement bias.

Two reviewers independently evaluated the certainty of evidence using GRADE, considering risk of bias, inconsistency, indirectness, imprecision, and publication bias (Guyatt et al., 2008). Disagreements were resolved by consensus and, when necessary, by consultation with a third reviewer. Certainty was rated as high, moderate, low, or very low for each clinically prioritized outcome.

### Statistical analysis

Binary outcomes were summarized as RRs with 95% confidence intervals (CIs). For adverse outcomes, an RR below 1 favored probiotics; for eradication and adherence outcomes, an RR above 1 favored probiotics. Random-effects models were used because clinical heterogeneity was anticipated across probiotic preparations, background regimens, patient populations, and treatment durations. Study-specific log RRs were pooled by inverse-variance weighting, between-study variance was estimated by restricted maximum likelihood, and Hartung-Knapp adjustments were applied to the CIs. Heterogeneity was assessed using Cochran’s Q, tau-squared, and I^2^. When at least three studies were available, 95% prediction intervals were calculated to describe the range of effects that might be expected in a future clinically comparable study (Riley et al., 2011; IntHout et al., 2014). Studies with zero events in both groups were summarized descriptively and did not contribute to an RR estimate. Individual and pooled effects were displayed in forest plots, and summary tables reported relative effects, absolute effects, prediction intervals, and certainty ratings.

Relative effects were translated into absolute effects per 1,000 participants using the pooled observed control-group risk. The primary overall-adverse-event analysis was restricted to studies with unambiguous participant-level data. Sensitivity analyses examined the influence of studies with unresolved reporting concerns, possible quasi-random allocation, an atypical 4-week eradication course, and dissertation-only reporting. Formal subgroup analyses and meta-regression were not performed because the number of contributing studies was insufficient for reliable effect-modifier analysis and most probiotic formulations were represented by only one study. Under these conditions, subgroup estimates would largely reflect individual-study effects and meta-regression would be highly unstable. Funnel plots and regression tests for small-study effects were also not performed because too few studies were available for reliable inference. Two-sided P values below 0.05 were considered statistically significant; however, interpretation prioritized effect magnitude, confidence and prediction intervals, event counts, sensitivity analyses, and GRADE certainty rather than statistical significance alone. Analyses were performed in R version 4.5.3 using the meta package.

## Results

### Study selection

The searches identified 506 records from bibliographic databases and 19 records from ClinicalTrials.gov. After 282 duplicate records were removed, 243 unique records underwent title and abstract screening, of which 219 were excluded. Twenty-four reports were retrieved and assessed for eligibility. One randomized trial was excluded because all participants were adolescents (Kakiuchi et al., 2020), and seven reports described ongoing studies or studies awaiting results. Sixteen reports corresponding to nine independent randomized studies were included in the systematic review (Figure 1). Additional searches of Chinese and WHO trial registries identified linked, ongoing, or awaiting-results registrations but no additional completed eligible trial with usable numerical outcome data.

**FIGURE 1:**
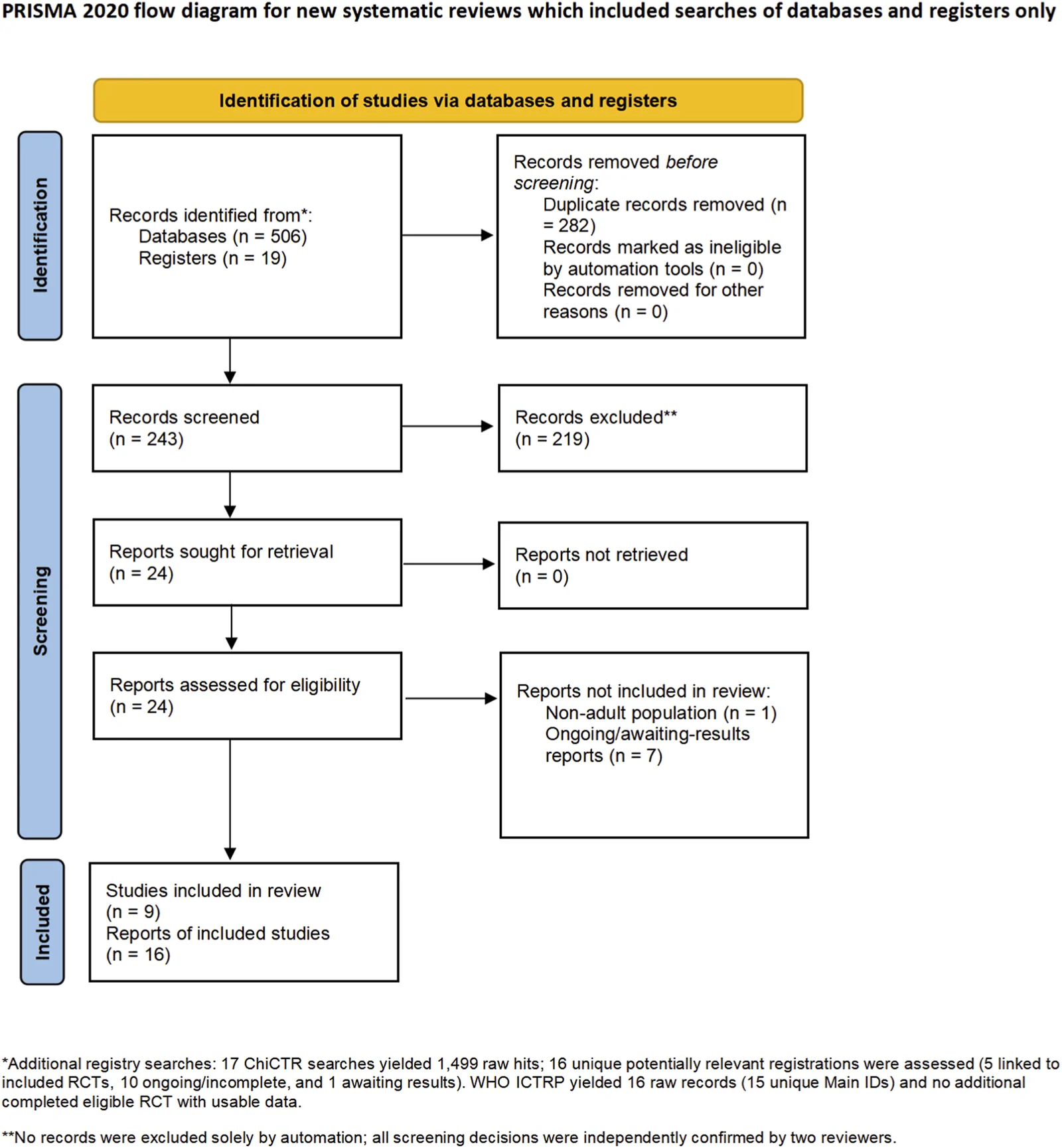
PRISMA 2020 flow diagram of study identification, screening, eligibility assessment, and inclusion.

### Characteristics of included studies

The nine included studies randomized 1,270 participants and were published or completed between 2023 and 2026 (Fan et al., 2026; Yang et al., 2026; Qu et al., 2026; Ji et al., 2026; Cheng and Wang, 2023; Zhao and Tan, 2023; Guo et al., 2025; Zhang, 2024; Dong, 2023). All studies were conducted in China; one was multicenter and eight were single-center. Seven studies were reported as journal articles and two as master’s dissertations. Six studies evaluated vonoprazan-amoxicillin dual therapy, one evaluated vonoprazan-based triple therapy, and two evaluated bismuth-containing quadruple therapy. Treatment lasted 10 days in two studies, 14 days in six studies, and 4 weeks in one atypical study. The probiotic interventions included combinations of *Bacillus subtilis* and *Enterococcus* faecium, *Saccharomyces* boulardii, compound lactic acid bacteria, viable Bifidobacterium-containing capsules, *Bacillus* licheniformis, and a Bifidobacterium tetravaccine. Most studies enrolled treatment-naive adults; one enrolled patients receiving rescue therapy, one focused on peptic ulcer disease, and one enrolled adults aged 60 years or older. Eradication was generally confirmed by 13C- or 14C-urea breath testing at least 4 weeks after treatment. Within each trial, the vonoprazan-based background regimen was identical between randomized groups; across trials, antibiotic backbones, bismuth use, treatment duration, and probiotic products varied (Table 1).

**TABLE 1:** Characteristics of included randomized studies.

Study	Design/population	N	Background regimen	Probiotic and comparator	Duration/eradication assessment	Key outcomes and methodological notes
Fan et al. (2026) (ST01)	5-Center open-label RCT; predominantly treatment-naive adults, 18–65 years	220	Vonoprazan 20 mg bid + amoxicillin 1,000 mg tid	Medilac-S (*B. subtilis* + E. faecium), 2 capsules tid vs. identical dual therapy	14 days; 13C-UBT>=4 weeks	ITT/mITT/PP eradication, overall AE, adherence. ChiCTR2400082446; overall AE used actual-treatment denominators
Yang et al. (2026) (ST03)	Single-center double-blind matched-placebo RCT; treatment-naive adults, 18–70 years	60	VA dual therapy; reported doses internally inconsistent	Live bacterial preparation 500 mg qid for 28 days vs. matched placebo	Eradication therapy: 14 days; 13C-UBT at 8 weeks	ITT/PP eradication, AE, GSRS, gastric/gut microbiota. Major internal inconsistencies; excluded from primary analyses
Qu et al. (2026) (ST04)	Single-center open-label RCT; rescue after >=1 prior failure	190	Vonoprazan 20 mg bid + amoxicillin 750 mg tid	S. boulardii 250 mg bid vs. identical dual therapy	14 days; 13C-UBT 4–6 weeks	ITT/PP eradication, adherence, AE. ChiCTR2300075382; overall-AE total ambiguous and excluded from primary AE analysis
Ji et al. (2026) (ST05)	Single-center open-label RCT; treatment-naive adults, 18–70 years	150	Vonoprazan 20 mg bid + amoxicillin 750 mg tid	S. boulardii 250 mg bid vs. identical dual therapy	10 days; 13C-UBT 4–6 weeks	ITT/mITT/PP eradication, overall AE, adherence, serious AE. ChiCTR2300075347
Cheng and Wang (2023) (ST07)	Single-center trial; allocation by clinic-attendance order; mainly first-line, 18–70 years	300	Vonoprazan 20 mg bid + amoxicillin 1 g tid	Compound lactic acid bacteria 1 g tid vs. identical dual therapy	10 days; 13C-UBT>=4 weeks	Eradication, overall/individual AE. Potentially quasi-random allocation and replacement of withdrawals; excluded from the primary analyses
Zhao and Tan (2023) (ST08)	Single-center random-number-table RCT; *H. pylori*-related peptic ulcer	70	Vonoprazan triple therapy with amoxicillin and clarithromycin	Enteric-coated viable Bifidobacterium capsules 0.35 g bid vs. identical triple therapy	4 weeks; 14C-UBT after treatment	Eradication, AE, recurrence. Atypical 4-week regimen; evaluated in sensitivity analyses
Guo et al. (2025) (ST09)	Single-center random-number-table RCT; treatment-naive adults	100	Vonoprazan-amoxicillin-clarithromycin-potassium bismuth citrate quadruple therapy	S. boulardii 0.5 g bid vs. identical quadruple therapy	14 days; breath test after treatment	Eradication, overall/individual AE, recurrence. Allocation concealment was not reported; the trial was unregistered
Zhang (2024) (ST10)	Single-center four-arm RCT in a master’s thesis; eligible B and D arms only; adults>=60 years	80	Vonoprazan bismuth quadruple therapy	B. licheniformis 0.5 g tid vs. identical quadruple therapy	14 days; 13C/14C-UBT 1 month	ITT/PP eradication, overall/individual AE. Only the eligible arms with identical background treatment were included
Dong (2023) (ST11)	Single-center double-blind matched-placebo RCT; treatment-naive or failure >=6 months earlier, 18–65 years	100	Vonoprazan 20 mg bid + amoxicillin 750 mg qid	Bifidobacterium tetravaccine 3 tablets tid vs. starch placebo	14 days; 13C-UBT 4 weeks	ITT/mITT/PP eradication, adherence, individual AE, gut/oral microbiota. ChiCTR2200060023; no participant-level count of overall adverse events

Abbreviations: AE, adverse event; bid, twice daily; GSRS, gastrointestinal symptom rating scale; ITT, intention-to-treat; mITT, modified intention-to-treat; PP, per-protocol; qid, four times daily; RCT, randomized controlled trial; tid, three times daily; UBT, urea breath test; VA, vonoprazan-amoxicillin. Within each study, the background vonoprazan regimen was identical between randomized groups; background regimens varied across studies.

Two companion reports evaluated the oral microbiota in subsets of the ST01 and ST11 populations (Wei et al., 2026; Peng et al., 2023). Additional gastric or intestinal microbiome data were available from ST03 and the ST11 dissertation. Sampling sites, assessment time points, diversity indices, sequencing pipelines, and reporting formats differed substantially; therefore, microbiome findings were summarized narratively rather than pooled (Supplementary Table S4).

### Risk of bias

All five studies contributing to the primary overall-adverse-event analysis were judged to have some concerns (Figure 2A). The principal concerns were open-label ascertainment of subjective adverse events, incomplete reporting of allocation concealment or prespecified analyses, and use of an actual-treatment or modified intention-to-treat denominator in one study. For intention-to-treat eradication, two of seven studies were judged to be at low risk of bias and five had some concerns (Figure 2B). Objective urea breath testing and limited missing outcome data reduced concerns about outcome measurement. ST03 and ST07 were judged to be at high risk because of major internal inconsistencies or potentially quasi-random allocation with replacement of withdrawals; both were excluded from the primary analyses. ST04 was excluded from the primary overall-adverse-event analysis because the reported total could not be verified as the number of unique participants with at least one event. ST11 did not report a separate participant-level count for overall adverse events. Detailed domain-level judgments are presented in Supplementary Figures S9,S10.

**FIGURE 2:**
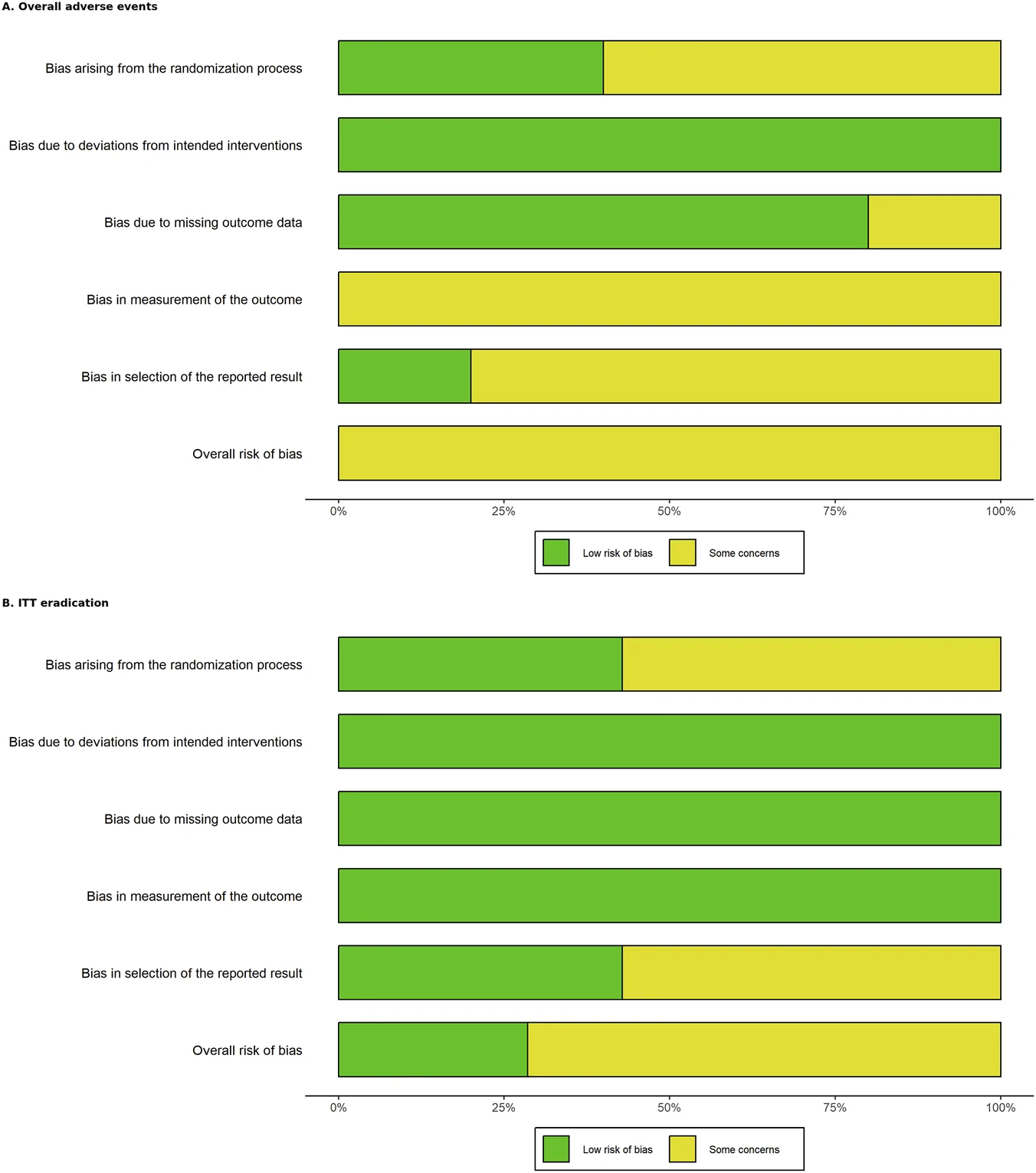
Outcome-specific Risk of Bias 2 summary plots for **(A)** overall adverse events and **(B)** intention-to-treat eradication.

### Overall adverse events

Five studies involving 611 participants provided unambiguous participant-level data for overall adverse events. The pooled effects for the clinically prioritized outcomes are summarized in Table 2. Ascertainment procedures varied across studies: two trials used structured participant questionnaires or recording forms, one used twice-weekly telephone follow-up, and two reported treatment-period adverse reactions without clearly describing the ascertainment method. Most assessments were unblinded or did not report blinding, which is relevant because adverse-event outcomes were largely subjective (Supplementary Table S6). Adjunctive probiotics reduced the risk of experiencing at least one adverse event (RR = 0.30, 95%CI: 0.17–0.53; P = 0.004; I^2^ = 0%; Figure 3). The 95% prediction interval (0.12–0.79) remained entirely below the null, suggesting that a benefit would also be expected in a future clinically comparable study. At the pooled control risk of 261 per 1,000, the estimate corresponded to 182 fewer participants experiencing an adverse event per 1,000 treated (95%CI: 122–216 fewer), equivalent to approximately one additional participant avoiding an adverse event for every six treated. The certainty of evidence was moderate because of outcome-specific risk-of-bias concerns.

**TABLE 2:** Summary of pooled effects for clinically prioritized outcomes.

Outcome	Studies (participants)	RR (95% CI)	P Value	I2	95% prediction interval	Absolute effect per 1,000	GRADE	Clinical interpretation
Overall adverse events	5 (611)	0.30 (0.17–0.53)	0.004	0.0%	0.12–0.79	182 fewer (122–216 fewer)	Moderate	Probably reduces the risk of experiencing at least one adverse event
*H. pylori* eradication (ITT)	7 (910)	1.09 (1.00–1.19)	0.044	47.8%	0.92–1.30	79 more (3–154 more)	Low	A small benefit is possible, but the finding is borderline and not robust
*H. pylori* eradication (PP)	5 (698)	1.03 (0.97–1.09)	0.263	31.4%	0.97–1.09	26 more (29 fewer to 85 more)	Low	Probably little or no effect on eradication success
Good adherence	3 (551)	1.03 (1.00–1.06)	0.056	0.0%	0.96–1.10	27 more (2 fewer to 47 more)	Low	Little or no effect, or at most a small improvement
Discontinuation due to AE	2 (285)	0.31 (0.00–836.60)	0.311	0.0%	Not interpretable	24 fewer (35 fewer to 965 more)	Very low	The effect is extremely uncertain because only six events occurred
Diarrhoea	7 (896)	0.44 (0.15–1.26)	0.104	0.0%	0.13–1.43	21 fewer (32 fewer to 10 more)	Very low	The effect is very uncertain
Serious adverse events	1 (150)	Not estimable (0/75 vs. 0/75)	—	—	—	No estimable difference	Very low	Zero events do not establish equivalent safety

Random-effects models used inverse-variance weighting, restricted maximum-likelihood estimation of between-study variance, and Hartung-Knapp confidence intervals. For adverse outcomes, an RR, below 1 favors probiotics; for eradication and adherence outcomes, an RR, above 1 favors probiotics. Absolute effects were calculated using the pooled observed control-group risk.

**FIGURE 3:**
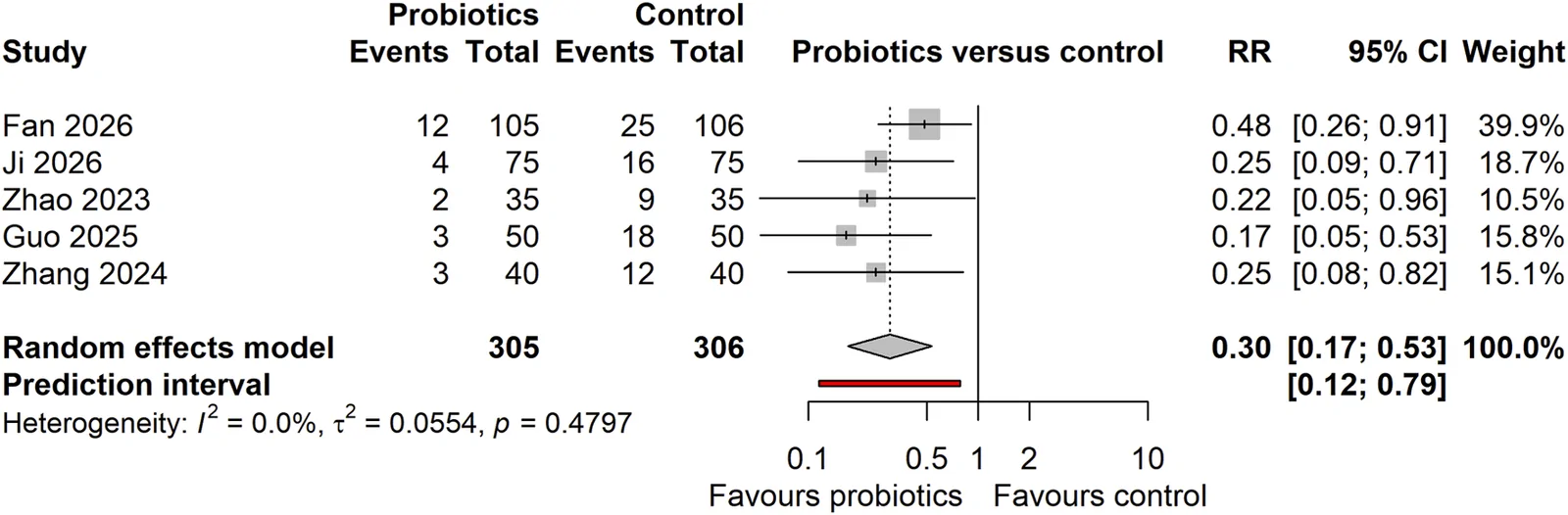
Random-effects meta-analysis of overall adverse events. Risk ratios below 1 favor adjunctive probiotics. The diamond represents the Hartung-Knapp random-effects estimate, and the horizontal bar below it represents the 95% prediction interval.

### *Helicobacter pylori* eradication efficacy

Seven studies involving 910 participants contributed to the primary intention-to-treat eradication analysis. The pooled estimate suggested a small increase in eradication with adjunctive probiotics (RR = 1.09, 95%CI: 1.00–1.19; P = 0.044; I^2^ = 47.8%; Figure 4), corresponding to 79 additional eradications per 1,000 treated at the pooled control risk. However, the lower confidence limit was close to the null, and the 95% prediction interval crossed 1.0 (0.92–1.30), indicating that a future clinically comparable study might show no benefit. The certainty of evidence was low.

**FIGURE 4:**
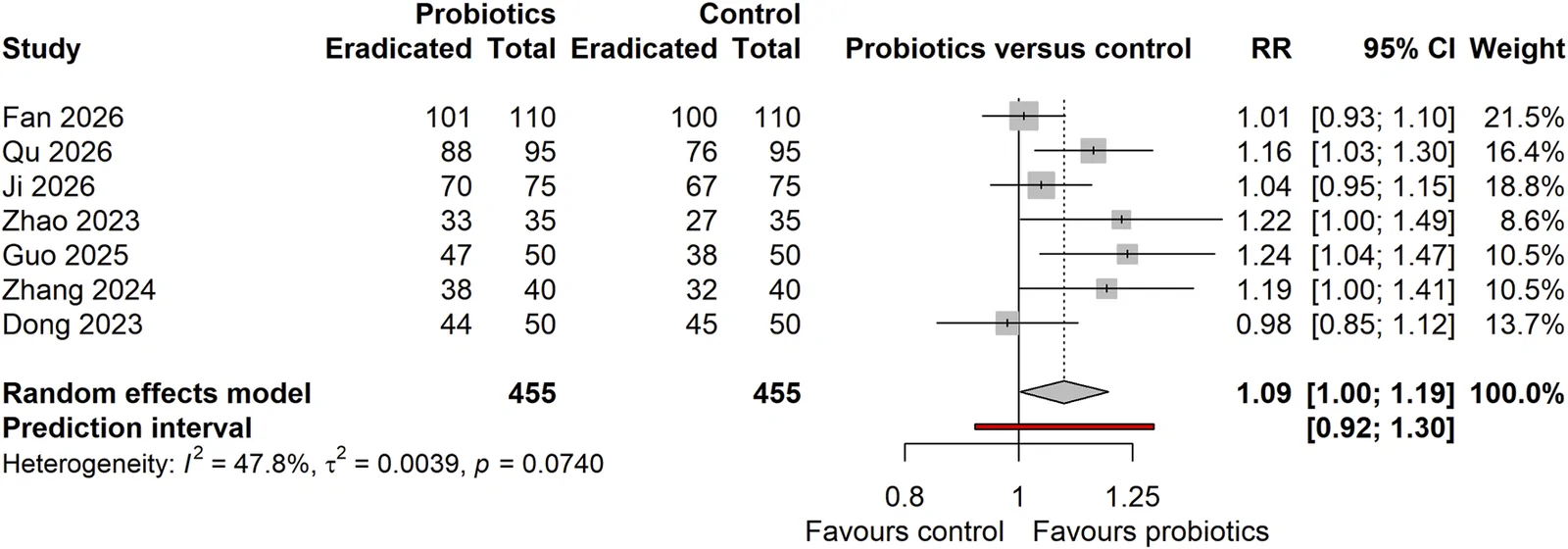
Random-effects meta-analysis of *Helicobacter pylori* eradication in the intention-to-treat population. Risk ratios above 1 favor adjunctive probiotics. The 95% prediction interval crossed 1.0, indicating uncertainty about the effect expected in a future clinically comparable study.

Five studies involving 698 participants contributed to the per-protocol analysis. The pooled estimate indicated little or no improvement in eradication (RR = 1.03, 95%CI: 0.97–1.09; P = 0.263; I^2^ = 31.4%; 95% prediction interval: 0.97–1.09; Supplementary Figure S1). This corresponded to 26 additional eradications per 1,000 treated, with the 95%CI ranging from 29 fewer to 85 more. The certainty of evidence was low.

### Adherence and treatment discontinuation

Three studies involving 551 participants reported good adherence. Adjunctive probiotics did not clearly improve adherence (RR = 1.03, 95%CI: 1.00–1.06; P = 0.056; I^2^ = 0%; 95% prediction interval: 0.96–1.10; Supplementary Figure S2). The control-group adherence rate was already high (953 per 1,000), limiting the potential absolute improvement. The certainty of evidence was low.

Two studies involving 285 participants reported discontinuation because of adverse events. Only six events occurred, resulting in an extremely imprecise estimate (RR = 0.31, 95%CI: 0.00–836.60; Supplementary Figure S3). The effect on treatment discontinuation therefore remained very uncertain. One study reported no serious adverse events in either group (0/75 vs. 0/75); this finding did not establish equivalent safety and was graded as very-low-certainty evidence.

### Individual adverse events

Seven studies involving 896 participants reported diarrhea. The pooled estimate favored probiotics but remained compatible with no difference or possible harm (RR = 0.44, 95%CI: 0.15–1.26; I^2^ = 0%; Supplementary Figure S4). Similar uncertainty was observed for abdominal pain (RR = 0.63, 95%CI: 0.13–3.12; Supplementary Figure S5). Pooled estimates suggested fewer episodes of constipation (RR = 0.29, 95%CI: 0.10–0.88), bloating (RR = 0.46, 95%CI: 0.23–0.93), and nausea (RR = 0.31, 95%CI: 0.17–0.57) (Supplementary Figures S6–S8). However, these analyses included few events, the prediction intervals crossed the null when calculable, and all were graded as very-low-certainty exploratory evidence (Supplementary Table S1).

### Sensitivity analyses and certainty of evidence

The overall-adverse-event finding was robust to alternative inclusion decisions. The pooled RR remained between 0.31 and 0.38 after exclusion of the atypical 4-week study, inclusion of studies with unresolved outcome or randomization concerns, and inclusion of all extractable data; statistical heterogeneity remained negligible (Supplementary Table S2). In contrast, the intention-to-treat eradication finding was not robust. Excluding dissertation-based studies yielded an RR of 1.10 (95%CI: 0.99–1.23; P = 0.071; I^2^ = 52.2%), whereas the strict analysis excluding the atypical 4-week study and both dissertations yielded an RR of 1.09 (95%CI: 0.95–1.25; P = 0.149; I^2^ = 55.4%) (Supplementary Table S3).

GRADE certainty was moderate for overall adverse events; low for intention-to-treat eradication, per-protocol eradication, and adherence; and very low for discontinuation, diarrhea, serious adverse events, and the remaining individual adverse events (Table 3).

**TABLE 3:** GRADE summary of findings.

Outcome	Studies (participants)	Control risk	Probiotic risk (95% CI)	Relative effect	Absolute effect	Certainty	Plain-language conclusion
Overall adverse events	5 (611)	261/1,000	79/1,000 (45–139)	RR 0.30 (0.17–0.53)	182 fewer/1,000 (122–216 fewer)	Moderate	Adjunctive probiotics probably reduce overall adverse events
Discontinuation due to AE	2 (285)	35/1,000	11/1,000 (0–1,000)	RR 0.31 (0.00–836.60)	24 fewer/1,000 (35 fewer to 965 more)	Very low	The effect on discontinuation is extremely uncertain
Good adherence	3 (551)	953/1,000	980/1,000 (951–1,000)	RR 1.03 (1.00–1.06)	27 more/1,000 (2 fewer to 47 more)	Low	There is probably little or no effect, or at most a small improvement
ITT eradication	7 (910)	846/1,000	925/1,000 (849–1,000)	RR 1.09 (1.00–1.19)	79 more/1,000 (3–154 more)	Low	A small benefit is possible, but the finding is borderline and not robust
PP eradication	5 (698)	913/1,000	939/1,000 (884–998)	RR 1.03 (0.97–1.09)	26 more/1,000 (29 fewer to 85 more)	Low	There is probably little or no effect on eradication success
Diarrhoea	7 (896)	38/1,000	17/1,000 (6–48)	RR 0.44 (0.15–1.26)	21 fewer/1,000 (32 fewer to 10 more)	Very low	The effect on diarrhea is very uncertain
Serious adverse events	1 (150)	0/75	0/75	Not estimable	Not estimable	Very low	Zero events cannot establish equivalent safety

GRADE, ratings: Moderate certainty indicates that the true effect is probably close to the estimate but may differ substantially; low certainty indicates that the true effect may differ substantially; very low certainty indicates very limited confidence in the estimate. Detailed reasons for downgrading are provided in the supplementary material.

## Discussion

### Principal findings

The clearest and most credible benefit of adjunctive probiotics was improved tolerability rather than a reliable increase in eradication success. The risk of experiencing at least one adverse event was approximately 70% lower, the absolute reduction was clinically meaningful, statistical heterogeneity was absent, the prediction interval supported benefit, and sensitivity analyses yielded consistent results. By contrast, the apparent intention-to-treat eradication benefit was modest, borderline, and sensitive to study selection. Per-protocol eradication showed no clear improvement, and adherence was already high and changed little. These findings distinguish a reasonably robust tolerability signal from an uncertain efficacy signal. Importantly, the moderate-certainty evidence for the participant-level outcome of experiencing at least one adverse event should not be extrapolated to individual adverse-event categories. The latter were supported by fewer events and were generally rated as very-low-certainty evidence.

### Clinical interpretation

The estimated absolute reduction in overall adverse events, from 261 to 79 per 1,000 participants, may be meaningful to patients and clinicians, particularly when gastrointestinal symptoms threaten treatment completion. At the pooled baseline risk, approximately one additional participant would avoid an adverse event for every six treated with adjunctive probiotics. This estimate should not be assumed to apply uniformly to every probiotic product because the trials evaluated biologically distinct bacterial and yeast preparations. Accordingly, the pooled estimate should be interpreted as the average effect of an adjunctive probiotic strategy across the included trials rather than evidence that all probiotic strains or formulations confer equivalent benefit. Nevertheless, the consistent direction of effect across products and sensitivity analyses supports improved tolerability as the most plausible clinical reason to consider adjunctive probiotics.

The eradication findings require greater caution. The control-group intention-to-treat eradication rate was already approximately 85%, leaving limited scope for absolute improvement. Although the pooled point estimate suggested a possible benefit, the prediction interval included no effect, and statistical significance was lost after exclusion of methodologically atypical or dissertation-based studies. The per-protocol estimate was close to the null. The nominal statistical significance of the primary intention-to-treat estimate should therefore not be interpreted as robust evidence that adjunctive probiotics improve eradication efficacy. Probiotics should therefore not be presented as a substitute for selecting an effective guideline-concordant eradication regimen, optimizing antibiotic exposure, supporting adherence, or confirming eradication after treatment. Their role is better framed as supportive care to improve tolerability.

### Why our findings differ from previous meta-analyses

A 2024 umbrella review of probiotic supplementation across heterogeneous *H. pylori* regimens reported higher eradication rates and fewer overall adverse effects but judged most of the underlying meta-analyses to be of critically low quality (Yang et al., 2024). Previous syntheses generally pooled proton pump inhibitor-, vonoprazan-, and other acid-suppression backgrounds while also combining different antibiotic backbones, bismuth use, probiotic strains, doses, and treatment durations. Our review addressed a narrower causal contrast: within each randomized comparison, the background vonoprazan regimen had to be identical, and probiotics were the principal treatment difference. This restriction reduces confounding by the underlying eradication regimen and may explain why the tolerability signal remained clear whereas the incremental eradication benefit was weaker and less robust than that reported in broader meta-analyses.

### Biological plausibility and microbiome evidence

Several mechanisms may contribute to improved tolerability, including attenuation of antibiotic-associated microbial disruption, enhancement of intestinal barrier function, competition with opportunistic organisms, production of antimicrobial metabolites, and modulation of mucosal immune responses. The limited microbiome data from the included trials were directionally compatible with faster recovery of selected diversity measures or beneficial taxa in some studies; however, findings differed by sampling site and probiotic product and could not be pooled. The clinical conclusions should therefore rest on the randomized adverse-event evidence rather than on an assumed universal microbiome mechanism.

### Strengths and limitations

This review has several strengths. It was prospectively registered, searched major English-language and Chinese-language databases and trial registries, linked multiple reports at the study level, and required identical background treatment within each randomized comparison. The integrity of the participant-level overall-adverse-event outcome was protected by excluding ambiguous cumulative event counts. Random-effects models used restricted maximum-likelihood estimation and Hartung-Knapp inference; prediction intervals were reported; and conclusions integrated RoB 2, GRADE, absolute effects, and prespecified sensitivity analyses. These safeguards were particularly important because most syntheses included few studies.

Several limitations warrant emphasis. First, all included trials were conducted in China; the findings should therefore not be assumed to generalize directly to other populations. Regional variation in *H. pylori* antimicrobial-resistance patterns, prior antibiotic exposure, dietary practices, baseline gastrointestinal microbiota, probiotic availability and product quality, healthcare delivery, and symptom-reporting behavior may modify both baseline risk and treatment effects. The geographic concentration of evidence is understandable because vonoprazan was adopted earlier and more widely in East Asia, but confirmation in diverse healthcare settings is required. Second, the evidence base was small, and probiotic products, strains, background regimens, populations, and treatment durations varied across trials. Several studies were open-label, and adverse events were subjectively reported, raising concerns about outcome measurement. Two studies were dissertations, one used an atypical 4-week eradication course, and two otherwise relevant studies were excluded from primary analyses because of serious reporting or allocation concerns. Rare outcomes were markedly underpowered, statistical assessment of publication bias was not possible, and the available evidence did not support meaningful subgroup analyses or meta-regression. Because most probiotic formulations were represented by only one trial, the available data could not reliably distinguish strain-specific treatment effects from individual-study effects. Finally, the data do not identify an optimal probiotic strain, dose, timing, or duration.

### Implications for practice and research

In clinical practice, adjunctive probiotics may be discussed with adults receiving vonoprazan-based eradication therapy when reducing treatment-related symptoms is a priority, provided that cost, availability, patient preference, contraindications, and product quality are considered. Because the included trials did not provide comparative economic evaluations, the cost-effectiveness of routine adjunctive probiotic use remains uncertain. Accordingly, these findings should not be interpreted as supporting universal probiotic supplementation for all patients receiving vonoprazan-based eradication therapy. The evidence does not support promising a reliably higher eradication rate. Clinicians should continue to prioritize an appropriate guideline-concordant regimen and confirm eradication after treatment.

Future trials should be adequately powered, prospectively registered, multicenter, and preferably placebo-controlled. They should use standardized participant-level adverse-event definitions, prespecified adherence thresholds, complete randomized denominators, and consistent timing of urea breath testing. Reports should specify probiotic strain identity, viable dose, timing relative to antibiotics, and product-quality controls. Head-to-head comparisons will be informative only after a sufficient evidence base has accumulated to support meaningful strain-level analyses. Core outcome sets and harmonized microbiome methods would improve comparability and enable mechanistic synthesis.

## Conclusion

Adjunctive probiotics probably reduce overall adverse events during vonoprazan-based *H. pylori* eradication therapy, with a clinically important absolute benefit and consistent sensitivity analyses. Their incremental effect on eradication success is small and uncertain, and adherence is not clearly improved. Probiotics should therefore be viewed primarily as supportive care for tolerability rather than as a reliable means of increasing eradication efficacy. Because all available trials were conducted in China, confirmation in other regions is required before broad global generalization.

## Data Availability

The original contributions presented in the study are included in the article/Supplementary Material, further inquiries can be directed to the corresponding author.

## References

[B1] Chen Y. C. Malfertheiner P. Yu H. T. Kuo C. L. Chang Y. Y. Meng F. T. (2024). Global prevalence of *Helicobacter pylori* infection and incidence of gastric cancer between 1980 and 2022. Gastroenterol 166, 605–619. 10.1053/j.gastro.2023.12.022 38176660

[B2] Cheng Y. J. Wang P. F. (2023). Effect of compound lactic acid bacteria-assisted dual therapy for *Helicobacter pylori* infection [in Chinese]. Acta Neuropharmacol. 13, 41–45. 10.3969/j.issn.2095-1396.2023.02.007

[B3] Chey W. D. Megraud F. Laine L. Lopez L. J. Hunt B. J. Howden C. W. (2022). Vonoprazan triple and dual therapy for *Helicobacter pylori* infection in the United States and Europe: randomized clinical trial. Gastroenterol 163, 608–619. 10.1053/j.gastro.2022.05.055 35679950

[B4] Chey W. D. Howden C. W. Moss S. F. Morgan D. R. Greer K. B. Grover S. (2024). ACG clinical guideline: treatment of *Helicobacter pylori* infection. Am. J. Gastroenterol. 119, 1730–1753. 10.14309/ajg.0000000000002968 39626064

[B5] Dong Y. (2023). Efficacy of probiotic-assisted vonoprazan-amoxicillin Dual Therapy for *Helicobacter pylori* Eradication and its Effect on Gut Microbiota. Nanjing: Nanjing Medical University.

[B6] Fan Y. Wang H. Yang Q. Niu C. Zhao J. Fei C. (2026). Vonoprazan-amoxicillin dual therapy combined with probiotics for *Helicobacter pylori* eradication: a prospective, multicenter, randomized controlled trial. BMC Gastroenterol. 26, 325. 10.1186/s12876-026-04820-7 41998531 PMC13218008

[B7] Guo D. Zhang Y. L. Zhuang K. M. (2025). Efficacy and safety of Saccharomyces boulardii-assisted vonoprazan quadruple therapy for *Helicobacter pylori* infection [in Chinese]. Chin. J. Integr. Trad. West Med. Dig. 33, 63–66. 10.3969/j.issn.1671-038X.2025.01.12

[B8] Guyatt G. H. Oxman A. D. Vist G. E. Kunz R. Falck‐Ytter Y. Alonso‐Coello P. (2008). GRADE: an emerging consensus on rating quality of evidence and strength of recommendations. BMJ 336, 924–926. 10.1136/bmj.39489.470347.AD 18436948 PMC2335261

[B9] Higgins J. P. T. Thomas J. Chandler J. Cumpston M. Li T. Page M. J. (2024). Cochrane Handbook for Systematic Reviews of Interventions Version 6.5. Available online at: https://www.cochrane.org/handbook .

[B10] Hu Y. Huang X. H. Zhou B. Liu M. L. Liu Y. F. Yu T. (2024). Vonoprazan and amoxicillin dual therapy for 14 days as the first-line treatment of *Helicobacter pylori* infection: a non-inferiority, randomized clinical trial. Helicobacter 29, e13045. 10.1111/hel.13045 39191423

[B11] IntHout J. Ioannidis J. P. A. Borm G. F. (2014). The hartung-knapp-sidik-jonkman method for random effects meta-analysis is straightforward and considerably outperforms the standard DerSimonian-Laird method. BMC Med. Res. Methodol. 14, 25. 10.1186/1471-2288-14-25 24548571 PMC4015721

[B12] Ji Z. Qu D. Feng Y. Qin X. Wang X. (2026). Effects of supplementing with Saccharomyces boulardii on vonoprazan-amoxicillin dual therapy for naive *Helicobacter pylori* infection: a randomized controlled trial. BMC Gastroenterol. 26, 347. 10.1186/s12876-026-04865-8 42026480 PMC13238084

[B13] Kakiuchi T. Mizoe A. Yamamoto K. Imamura I. Hashiguchi K. Kawakubo H. (2020). Effect of probiotics during vonoprazan-containing triple therapy on gut microbiota in *Helicobacter pylori* infection: a randomized controlled trial. Helicobacter 25, e12690. 10.1111/hel.12690 32207209

[B14] Malfertheiner P. Megraud F. Rokkas T. Gisbert J. P. Liou J. M. Schulz C. (2022). Management of *Helicobacter pylori* infection: the Maastricht VI/Florence consensus report. Gut 71, 1724–1762. 10.1136/gutjnl-2022-327745 35944925

[B15] Page M. J. McKenzie J. E. Bossuyt P. M. Boutron I. Hoffmann T. C. Mulrow C. D. (2021). The PRISMA 2020 statement: an updated guideline for reporting systematic reviews. BMJ 372, n71. 10.1136/bmj.n71 33782057 PMC8005924

[B16] Peng R. Zhang Z. Qu Y. Chen W. (2023). The impact of *Helicobacter pylori* eradication with vonoprazan-amoxicillin dual therapy combined with probiotics on oral microbiota: a randomized double-blind placebo-controlled trial. Front. Microbiol. 14, 1273709. 10.3389/fmicb.2023.1273709 37849923 PMC10577438

[B17] Qu D. N. Ji Z. X. Zhang J. Q. Wang X. Y. (2026). Vonoprazan-amoxicillin dual therapy with or without Saccharomyces boulardii supplementation as a rescue regimen for *Helicobacter pylori* infection. Infect. Drug Resist 19, 1–10. 10.2147/IDR.S594118 PMC13069978 41971394

[B18] Riley R. D. Higgins J. P. T. Deeks J. J. (2011). Interpretation of random effects meta-analyses. BMJ 342, d549. 10.1136/bmj.d549 21310794

[B19] Sterne J. A. C. Savovic J. Page M. J. Elbers R. G. Blencowe N. S. Boutron I. (2019). RoB 2: a revised tool for assessing risk of bias in randomised trials. BMJ 366, l4898. 10.1136/bmj.l4898 31462531

[B20] Wei W. Chen W. Peng R. Wang X. Wang Z. Zhang Z. (2026). Probiotics alleviate oral microbiota disruptions induced by *Helicobacter pylori* eradication with vonoprazan-amoxicillin dual therapy: a randomized double-blind placebo-controlled exploratory study. Front. Cell. Infect. Microbiol. 16, 1740968. 10.3389/fcimb.2026.1740968 42211655 PMC13212275

[B21] Yang Z. Zhou Y. Han Z. He K. Zhang Y. Wu D. (2024). The effects of probiotics supplementation on *Helicobacter pylori* standard treatment: an umbrella review of systematic reviews with meta-analyses. Sci. Rep. 14, 10069. 10.1038/s41598-024-59399-4 38697990 PMC11066092

[B22] Yang Y. Lan C. Jiang Z. (2026). Impact of probiotic-adjunctive vonoprazan-amoxicillin dual therapy on gut microbiota and clinical symptoms in *Helicobacter pylori* eradication. Tissue Eng. Regen. Med. 23, 653–666. 10.1007/s13770-026-00803-7 42018226 PMC13323411

[B23] Zhang B. (2024). Clinical study of Probiotics Combined with Vonoprazan Quadruple Therapy for *Helicobacter pylori* Infection in Elderly Patients [master's Thesis, in Chinese]. Xi'an: Xi'an Medical University.

[B24] Zhao H. N. Tan Y. M. (2023). Efficacy of Bifidobacterium viable enteric-coated capsules combined with *Helicobacter pylori* eradication therapy in *H. pylori*-related peptic ulcers [in Chinese]. Hainan Med. J. 34, 1989–1992. 10.3969/j.issn.1003-6350.2023.14.004

[B25] Zhou L. Lu H. Song Z. Lyu B. Chen Y. Wang J. (2022). Chinese national clinical practice guideline on *Helicobacter pylori* eradication treatment. Chin. Med. J. Engl. 135, 2899–2910. 10.1097/CM9.0000000000002546 36579940 PMC10106216

