# Supplementary material for: Adjunctive probiotics may reduce adverse events during vonoprazan-based Helicobacter pylori eradication: a systematic review and meta-analysis
Source: Front Pharmacol. 2026 Oct 19;17:1946885. doi: 10.3389/fphar.2026.1946885 (PMC13724370; doi:10.3389/fphar.2026.1946885)

# Supplementary Material

## Adjunctive probiotics may reduce adverse events during vonoprazan-based *Helicobacter pylori* eradication: A systematic review and meta-analysis

**Chun-Jiang Lin**[1], **Peng-Yuan Ren**[1], **Hong-Yu Chen**[1], **Ming-Qiao Zheng**[1], **Yong-Xian Jiang**[2], **Gen Li**[2], **Min Fu**[2], and **Ya-Hui Guo**[2*]

1 Department of Pharmacy, Second People's Hospital of Ya'an City, Ya'an, Sichuan, China

2 Department of Pharmacy, Sichuan Provincial Maternity and Child Health Care Hospital and The Affiliated Women's and Children's Hospital of Chengdu Medical College, Chengdu, Sichuan, China

*Correspondence: Ya-Hui Guo, 18728196538@163.com

## Supplementary Appendix 1. Search strategies

All searches were conducted without restrictions on publication date, language, study-design filter, participant-age filter, or publication status. The searches were performed on July 14-15, 2026. A chronological audit log and the original platform exports are retained in the review archive.

**PubMed - main probiotic search (34 records)**

("*Helicobacter pylori*"[Mesh] OR "*Helicobacter pylori*"[tiab] OR "*H. pylori*"[tiab] OR "H pylori"[tiab]) AND (vonoprazan[tiab] OR "TAK-438"[tiab] OR "TAK 438"[tiab] OR "potassium-competitive acid blocker"[tiab] OR "potassium competitive acid blocker"[tiab] OR "P-CAB"[tiab] OR PCAB[tiab]) AND ("Probiotics"[Mesh] OR probiotic*[tiab] OR microecolog*[tiab] OR "live microbial product"[tiab] OR "live microbial products"[tiab] OR "Saccharomyces boulardii"[tiab] OR Bifidobacter*[tiab] OR Lactobacill*[tiab] OR Lacticaseibacill*[tiab] OR Bacillus[tiab] OR Enterococcus[tiab] OR "Bacillus licheniformis"[tiab] OR "Clostridium butyricum"[tiab] OR "compound lactic acid bacteria"[tiab] OR "Medilac-S"[tiab])

**PubMed - supplementary microbiome search (27 records)**

("*Helicobacter pylori*"[Mesh] OR "*Helicobacter pylori*"[tiab] OR "*H. pylori*"[tiab] OR "H pylori"[tiab]) AND (vonoprazan[tiab] OR "TAK-438"[tiab] OR "TAK 438"[tiab] OR "potassium-competitive acid blocker"[tiab] OR "potassium competitive acid blocker"[tiab] OR "P-CAB"[tiab] OR PCAB[tiab]) AND (microbiota[tiab] OR microbiome*[tiab] OR microflora[tiab] OR dysbiosis[tiab] OR "microbial diversity"[tiab] OR "alpha diversity"[tiab] OR "beta diversity"[tiab] OR "16S rRNA"[tiab] OR metagenom*[tiab])

**Embase.com - main probiotic search (90 records)**

#1 'helicobacter pylori'/exp; #2 ('helicobacter pylori' OR 'h pylori' OR 'h. pylori'):ti,ab,kw; #3 #1 OR #2; #4 'vonoprazan'/exp; #5 (vonoprazan OR 'tak-438' OR 'tak 438' OR 'potassium competitive acid blocker' OR 'potassium-competitive acid blocker' OR 'p-cab' OR pcab):ti,ab,kw; #6 #4 OR #5; #7 'probiotic agent'/exp; #8 (probiotic* OR microecolog* OR 'live microbial product' OR 'live microbial products' OR 'saccharomyces boulardii' OR bifidobacter* OR lactobacill* OR lacticaseibacill* OR bacillus OR enterococcus OR 'bacillus licheniformis' OR 'clostridium butyricum' OR 'compound lactic acid bacteria' OR 'medilac-s'):ti,ab,kw; #9 #7 OR #8; #10 #3 AND #6 AND #9.

**Embase.com - supplementary microbiome search (73 records)**

#11 (microbiota OR microbiome* OR microflora OR dysbiosis OR 'microbial diversity' OR 'alpha diversity' OR 'beta diversity' OR '16s rrna' OR metagenom*):ti,ab,kw; #12 #3 AND #6 AND #11.

**CENTRAL (34 probiotic records; 42 microbiome records)**

#1 MeSH descriptor: [*Helicobacter pylori*] explode all trees; #2 ("*Helicobacter pylori*" OR "*H. pylori*" OR "H pylori"):ti,ab,kw; #3 #1 OR #2; #4 (vonoprazan OR "TAK-438" OR "TAK 438" OR "potassium-competitive acid blocker" OR "potassium-competitive acid blockers" OR "potassium competitive acid blocker" OR "potassium competitive acid blockers" OR "P-CAB" OR PCAB):ti,ab,kw; #5 (probiotic* OR microecolog* OR "live microbial" OR "live microbial product" OR "live microbial products" OR "*Saccharomyces boulardii*" OR Bifidobacter* OR Lactobacill* OR Bacillus OR Enterococcus OR "*Bacillus licheniformis*" OR "*Clostridium butyricum*"):ti,ab,kw; #6 #3 AND #4 AND #5 (34 records). Supplementary microbiome search: #7 (microbiota OR microbiome* OR microflora OR dysbiosis OR "microbial diversity" OR "alpha diversity" OR "beta diversity" OR "16S rRNA" OR metagenom*):ti,ab,kw; #8 #3 AND #4 AND #7 (42 records). Results were restricted to the Trials section of the Cochrane Library.

**Web of Science Core Collection, SCIE (41 probiotic records; 45 microbiome records)**

TS=("*Helicobacter pylori*" OR "*H. pylori*" OR "H pylori") AND TS=(vonoprazan OR "TAK-438" OR "TAK 438" OR "potassium-competitive acid blocker*" OR "potassium competitive acid blocker*" OR "P-CAB" OR PCAB) AND TS=(probiotic* OR microecolog* OR "live microbial product*" OR "Saccharomyces boulardii" OR Bifidobacter* OR Lactobacill* OR Lacticaseibacill* OR Bacillus OR Enterococcus OR "Bacillus licheniformis" OR "Clostridium butyricum" OR "compound lactic acid bacteria" OR "Medilac-S"). The microbiome set substituted microbiota/microbiome/microflora/dysbiosis/diversity/16S/metagenomic terms.

**Chinese bibliographic databases**

CNKI: Three topic-field searches combined (幽门螺杆菌+幽门螺旋杆菌) AND (伏诺拉生+富马酸伏诺拉生+沃诺拉赞+vonoprazan+TAK-438) with, respectively, (1) 益生菌+微生态制剂+活菌制剂; (2) 布拉氏酵母菌+双歧杆菌+乳酸杆菌+复合乳酸菌+枯草杆菌+地衣芽孢杆菌+肠球菌+酪酸梭菌; or (3) 肠道菌群+口腔菌群+胃内菌群+微生物组+微生态+菌群多样性+16S+16S rRNA (33 records in total). Wanfang: Topic-field searches combined (幽门螺杆菌 OR 幽门螺旋杆菌) AND (伏诺拉生 OR 富马酸伏诺拉生 OR 沃诺拉赞 OR vonoprazan OR TAK-438) with either the named probiotic terms above (34 records) or the microbiome terms above (15 records). SinoMed/CBM: Common-field intelligent searches combined the corresponding *H. pylori* and vonoprazan terms with (1) generic probiotic terms (7 records), (2) named probiotic terms (10 records), or (3) microbiome terms (10 Chinese-language records). VIP: Title-or-keyword searches combined the corresponding *H. pylori* and vonoprazan terms with (1) generic probiotic terms (2 records), (2) named probiotic terms (4 records), or (3) microbiome terms (5 records). Platform-specific syntax was used without changing the prespecified concepts.

**ClinicalTrials.gov**

Three unrestricted ClinicalTrials.gov searches were conducted: (1) condition "*Helicobacter pylori*" with other terms "vonoprazan AND (probiotic OR probiotics OR microbiota OR

microbiome)" (10 records); (2) condition "*Helicobacter pylori*" with vonoprazan and named probiotic organisms or products (5 records); and (3) condition "*Helicobacter pylori*" with TAK-438, P-CAB, or potassium-competitive acid blocker combined with probiotic, microbiota, or microbiome terms (4 records). Nineteen raw records were retrieved, and 11 unique NCT records remained after within-register deduplication.

**ChiCTR and WHO ICTRP**

ChiCTR registration-title searches used 17 prespecified single terms covering vonoprazan/P-CAB synonyms and probiotic, named-product, live-bacteria, microecology, and microbiota concepts. The searches generated 1,499 raw result occurrences. After on-platform screening and deduplication by registration number, 16 potentially relevant unique registrations underwent detailed assessment. WHO ICTRP searches were: "vonoprazan AND probiotic" (2 records), "vonoprazan AND microbiota" (11 records), "vonoprazan AND microbiome" (2 records), "P-CAB AND probiotic" (1 record), "TAK-438 AND probiotic" (0 records), and "potassium-competitive acid blocker AND probiotic" (0 records). The 16 raw WHO records represented 15 unique Main IDs after within-platform deduplication; cross-register duplicates were linked at the study level.

# Supplementary Tables

## Supplementary Table S1. Exploratory individual gastrointestinal adverse events

| Outcome | Studies (participants) | RR (95% CI) | P value | I² | Prediction interval | Absolute effect per 1,000 | GRADE | Interpretation |
|---|---|---|---|---|---|---|---|---|
| Abdominal pain | 4 (646) | 0.63 (0.13-3.12) | 0.424 | 0.0% | 0.12-3.24 | 16 fewer (38 fewer to 92 more) | Very low | The effect is very uncertain. |
| Constipation | 6 (826) | 0.29 (0.10-0.88) | 0.035 | 0.0% | 0.06-1.33 | 22 fewer (3-28 fewer) | Very low | A reduction is possible, but the evidence is based on few events and remains very uncertain. |
| Bloating | 5 (726) | 0.46 (0.23-0.93) | 0.031 | 0.0% | 0.14-1.45 | 28 fewer (4-40 fewer) | Very low | A reduction is possible, but the prediction interval crosses the null. |
| Nausea | 4 (515) | 0.31 (0.17-0.57) | 0.008 | 0.0% | 0.05-2.19 | 30 fewer (19-36 fewer) | Very low | Possible reduction; only 14 events. |

Supplementary Table S2. Sensitivity analyses for overall adverse events

| Analysis | Studies | RR (95% CI) | $I^2$ | Interpretation |
|---|---|---|---|---|
| Primary analysis | ST01, ST05, ST08, ST09, ST10 | 0.302 (0.171-0.532) | 0.0% | The reduction was robust. |
| Excluding atypical 4-week regimen | ST01, ST05, ST09, ST10 | 0.309 (0.144-0.665) | 7.2% | The direction and statistical significance were unchanged. |
| Including ST03 | Primary + ST03 | 0.365 (0.229-0.580) | 0.5% | The direction and statistical significance were unchanged. |
| Including ST04 | Primary + ST04 | 0.330 (0.222-0.490) | 0.0% | The direction and statistical significance were unchanged. |
| Including ST07 | Primary + ST07 | 0.339 (0.223-0.516) | 0.0% | The direction and statistical significance were unchanged. |
| All extractable studies | All studies with extractable overall-AE data | 0.376 (0.283-0.499) | 0.0% | The direction and statistical significance were unchanged. |

Supplementary Table S3. Sensitivity analyses for ITT eradication

| Analysis | Studies/selection rule | RR (95% CI) | P value | $I^2$ | Interpretation |
|---|---|---|---|---|---|
| Primary | Seven eligible primary-analysis studies | 1.093 (1.003-1.191) | 0.044 | 47.8% | Borderline benefit. |
| Excluding dissertation-based studies | Excluded ST10 and ST11 | 1.104 (0.987-1.234) | 0.071 | 52.2% | The association was no longer statistically significant. |
| Strict sensitivity analysis | Excluded ST08, ST10, and ST11 | 1.088 (0.947-1.251) | 0.149 | 55.4% | No clear benefit was observed. |

Supplementary Table S4. Narrative summary of microbiome outcomes

| Study/report | Sampling site | Analysis sample/time points | Methods/metrics | Main finding | Quantitative synthesis |
|---|---|---|---|---|---|
| ST01 companion report | Oral tongue coating | Probiotic 31; VA 33; H. pylori-negative 20; weeks 0, 2, 6 | 16S V3-V4; Chao1, Shannon, Bray-Curtis, LEfSe, KEGG | Probiotic group showed smaller fluctuations in selected measures and partial attenuation of oral-community disruption. | Not pooled because incomplete continuous data and different comparators/metrics. |
| ST03 | Stool and gastric mucosa | 30 per group; days 0, 15, 29, 71 | 16S; alpha/beta diversity, LEfSe, taxa | Faster recovery of alpha diversity at day 29 and enrichment of selected butyrate-producing taxa were reported. | Not pooled because graphical reporting and internal inconsistencies. |
| ST11 dissertation | Stool | Probiotic 33; placebo 33; H. pylori-negative 22; weeks 0, 2, 6 | 16S; Chao1, Simpson, beta diversity, LEfSe, predicted function | Probiotics may have attenuated selected structural and functional disturbances after VA therapy. | Not pooled because continuous statistics incompletely reported. |
| ST11 companion report | Oral tongue coating | Probiotic 29; placebo 31; H. pylori-negative 20; weeks 0, 2, 6 | 16S V3-V4; Chao1, Shannon, PCoA, LEfSe, KEGG | No clear evidence that probiotic supplementation reduced oral microbiota disruption. | Not pooled because mainly figures and P values; same randomized population as dissertation. |

Supplementary Table S5. ChiCTR registrations retained after on-platform screening and deduplication

| Registration | Final classification | Link/notes |
|---|---|---|
| ChiCTR2200060023 | Linked to an included randomized study | Companion registry record; did not increase the independent study count. |
| ChiCTR2200064869 | Linked to an included randomized study | Companion registry record; did not increase the independent study count. |
| ChiCTR2300075347 | Linked to an included randomized study | Companion registry record; did not increase the independent study count. |
| ChiCTR2300075382 | Linked to an included randomized study | Companion registry record; did not increase the independent study count. |
| ChiCTR2400082446 | Linked to an included randomized study | Companion registry record; did not increase the independent study count. |
| ChiCTR2300075885 | Eligible study awaiting results | No numerical results publicly available; investigator contact did not yield data. |
| ChiCTR2400090233 | Ongoing or incomplete study | No usable randomized outcome data at the final search. |
| ChiCTR2500102908 | Ongoing or incomplete study | No usable randomized outcome data at the final search. |
| ChiCTR2500105392 | Ongoing or incomplete study | No usable randomized outcome data at the final search. |
| ChiCTR2600123681 | Ongoing or incomplete study | No usable randomized outcome data at the final search. |
| ChiCTR2600123827 | Ongoing or incomplete study | No usable randomized outcome data at the final search. |
| ChiCTR2600127614 | Ongoing or incomplete study | No usable randomized outcome data at the final search. |
| ChiCTR2200065864 | Ongoing or incomplete study | No usable randomized outcome data at the final search. |
| ChiCTR2500105014 | Ongoing or incomplete study | No usable randomized outcome data at the final search. |
| ChiCTR2500112845 | Ongoing or incomplete study | No usable randomized outcome data at the final search. |
| ChiCTR2500112879 | Ongoing or incomplete study | No usable randomized outcome data at the final search. |

Supplementary Table S6. Definition and ascertainment of overall adverse events in studies contributing to the primary analysis

| **Study** | Blinding | AE definition and ascertainment | Assessment period | Overall AE denominator | Reporting limitations |
|---|---|---|---|---|---|
| Fan et al. 2026 (ST01) | Open-label; no matched placebo | Participants recorded prespecified AEs using a structured electronic/paper form (bad breath, diarrhea, constipation, acid reflux/heartburn, nausea/vomiting, bloating, abdominal pain, dizziness/headache, and other symptoms). Study staff subsequently verified occurrence, severity, and potential relationship to medication by online/telephone follow-up. | Throughout 14-day therapy; verified within 3 days after completion | 12/105 vs 25/106; mITT denominators | Open-label subjective reporting; denominator excluded some randomized participants |
| Ji et al. 2026 (ST05) | Open-label | A standard questionnaire was used after therapy to record AEs and medication use. AEs were graded as mild, moderate, or severe according to interference with daily activities. | Following the 10-day treatment course; detailed within-treatment schedule not reported | 4/75 vs 16/75 | Open-label subjective assessment; ascertainment frequency not reported |
| Zhao et al. 2023 (ST08) | Not reported | Adverse reactions during treatment were recorded; reported categories included vomiting, diarrhea, taste disturbance, loss of appetite, and pseudomembranous colitis. The specific ascertainment method was not reported. | During 4-week treatment | 2/35 vs 9/35 | Blinding, ascertainment method/frequency, severity assessment, and handling of multiple symptoms were not clearly reported |

| **Study** | Blinding | AE definition and ascertainment | Assessment period | Overall AE denominator | Reporting limitations |
|---|---|---|---|---|---|
| Guo et al. 2025 (ST09) | Not reported | Adverse reactions during treatment were compared; reported categories included diarrhea, constipation, headache, urticaria, and drug dermatitis. The specific ascertainment method was not reported. | During 14-day treatment | 3/50 vs 18/50 | Blinding, ascertainment method/frequency, severity assessment, and handling of multiple symptoms were not clearly reported |
| Zhang, 2024 (ST10) | Not reported | Participant-level AE incidence was recorded by telephone follow-up; prespecified symptoms included loss of appetite, diarrhea, abdominal pain, nausea, vomiting, bloating, constipation, rash, and bitter taste. | During 14-day treatment; telephone follow-up twice weekly | Probiotic vs control: 3/40 vs 12/40; explicitly defined as participants with AE/total group size | No blinding reported; subjective telephone ascertainment |

AE, adverse event; mITT, modified intention-to-treat. “Not reported” indicates that the corresponding methodological detail was not clearly described in the source report.

# Supplementary Figures

## Supplementary Figure S1. Random-effects meta-analysis of per-protocol *H. pylori* eradication.

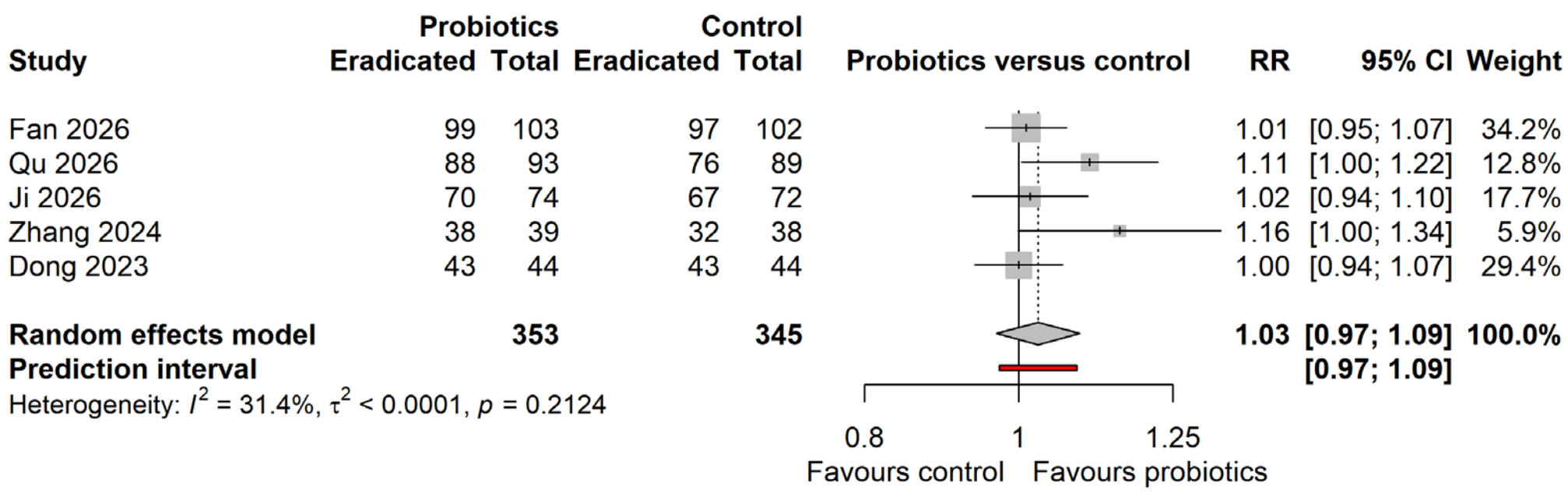

Supplementary Figure S2. Random-effects meta-analysis of good adherence.

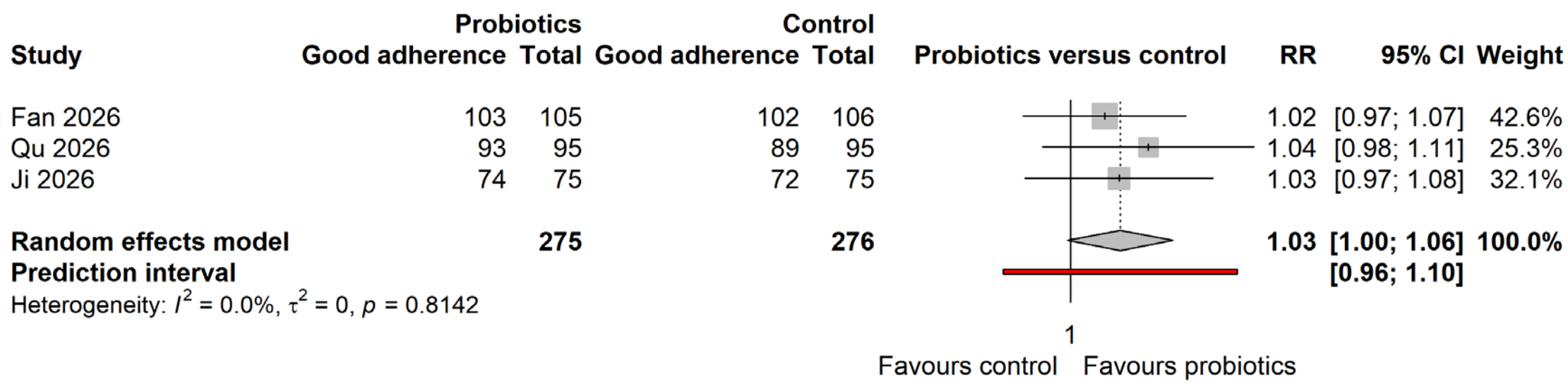

Supplementary Figure S3. Random-effects meta-analysis of discontinuation because of adverse events.

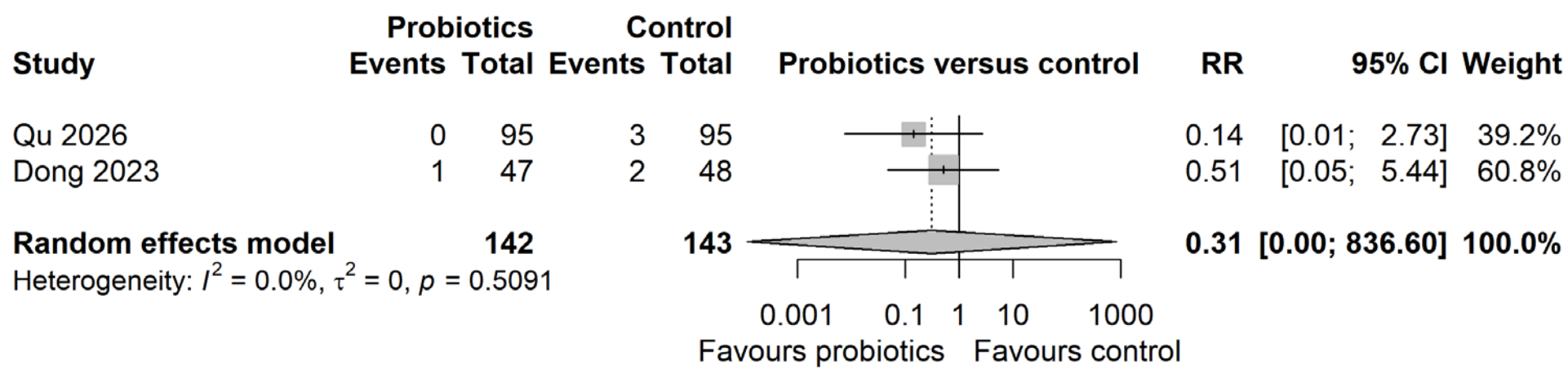

Supplementary Figure S4. Random-effects meta-analysis of diarrhea.

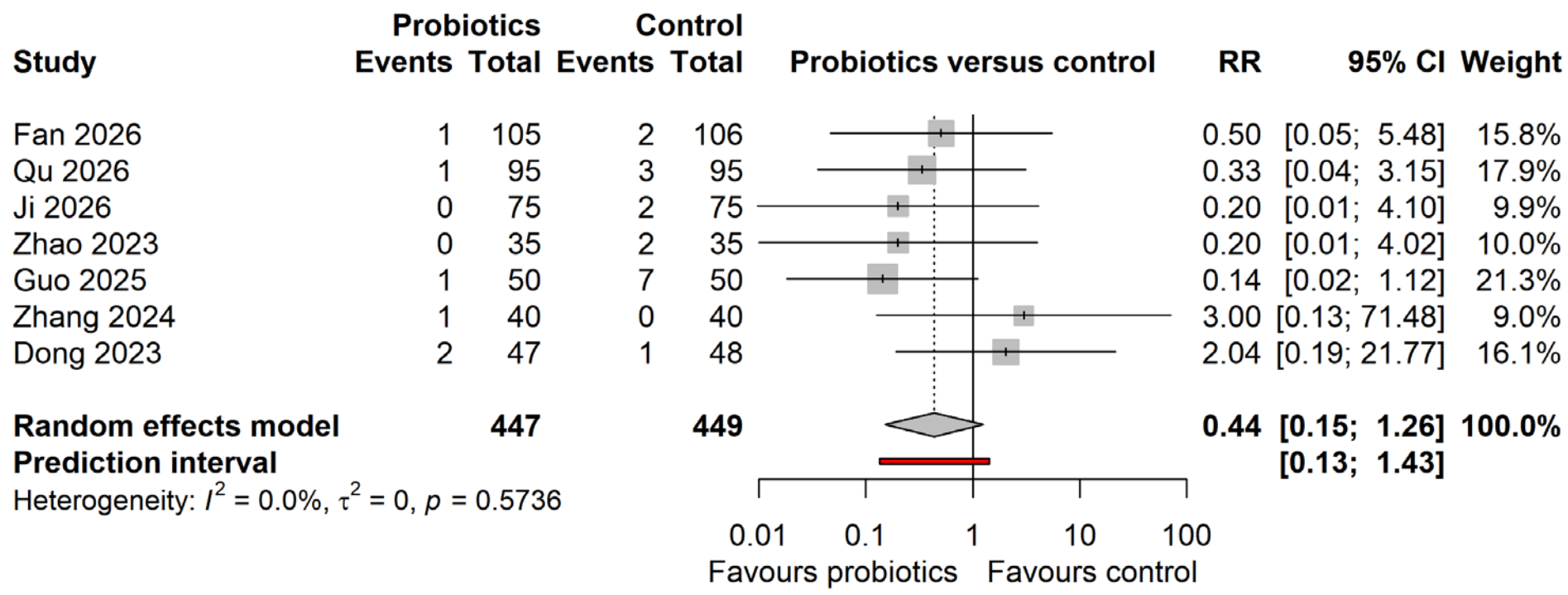

Supplementary Figure S5. Random-effects meta-analysis of abdominal pain.

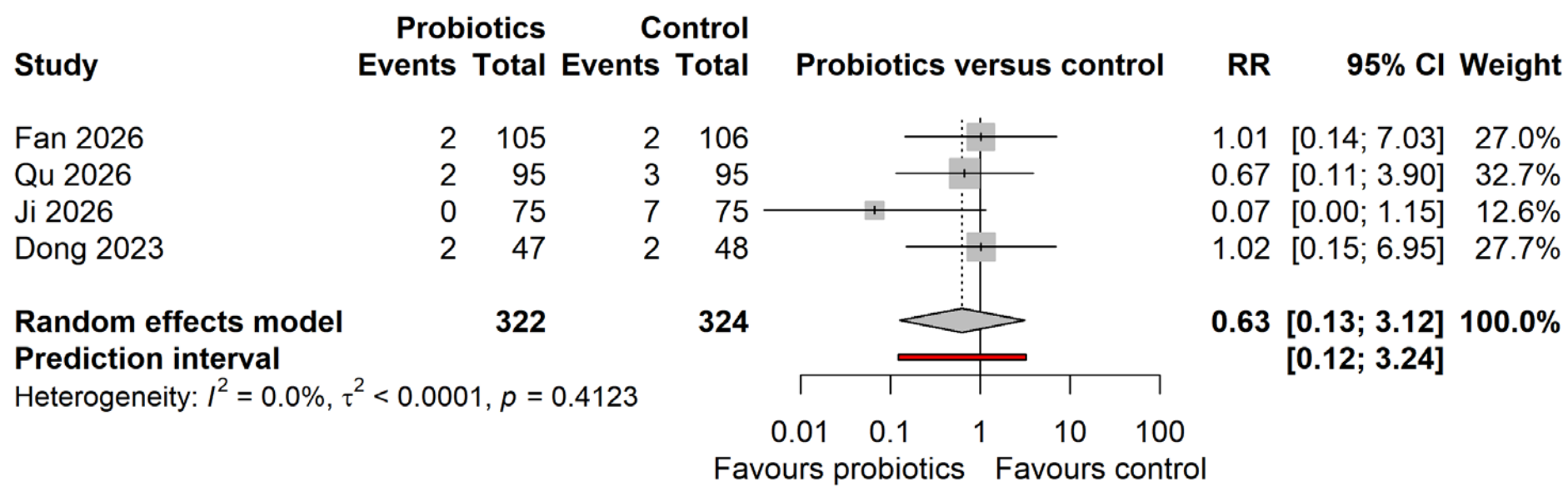

Supplementary Figure S6. Random-effects meta-analysis of constipation.

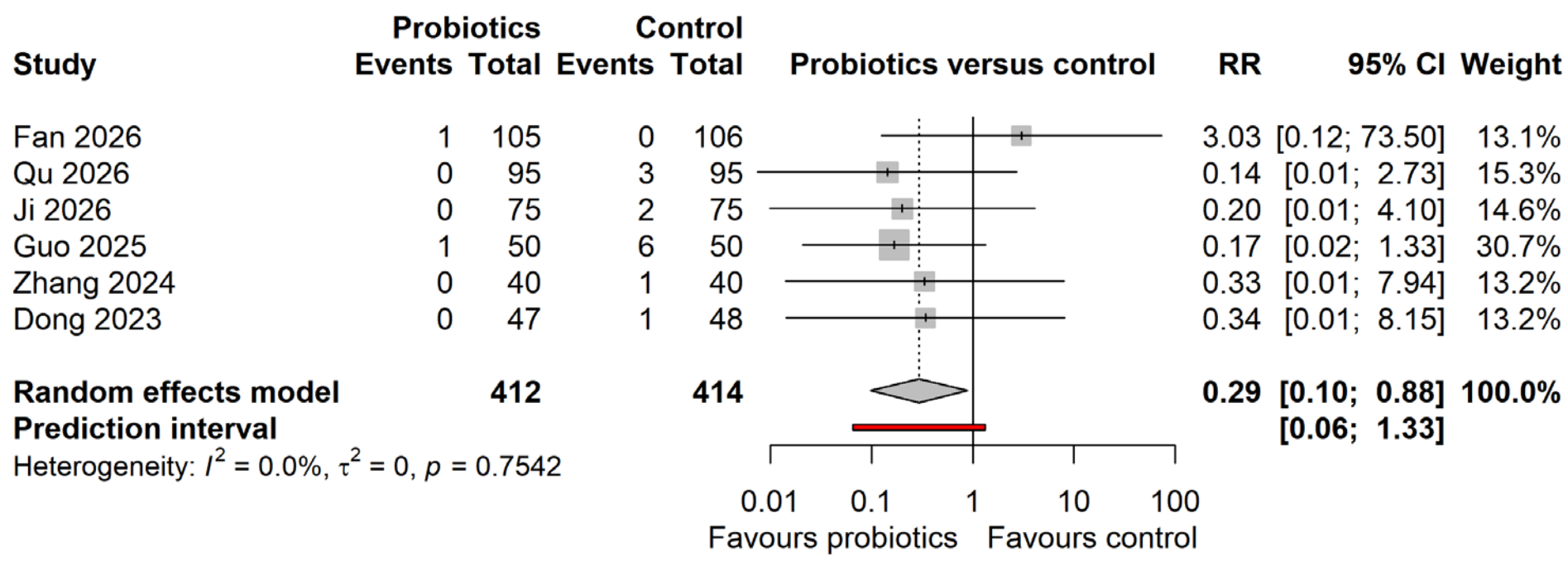

Supplementary Figure S7. Random-effects meta-analysis of bloating.

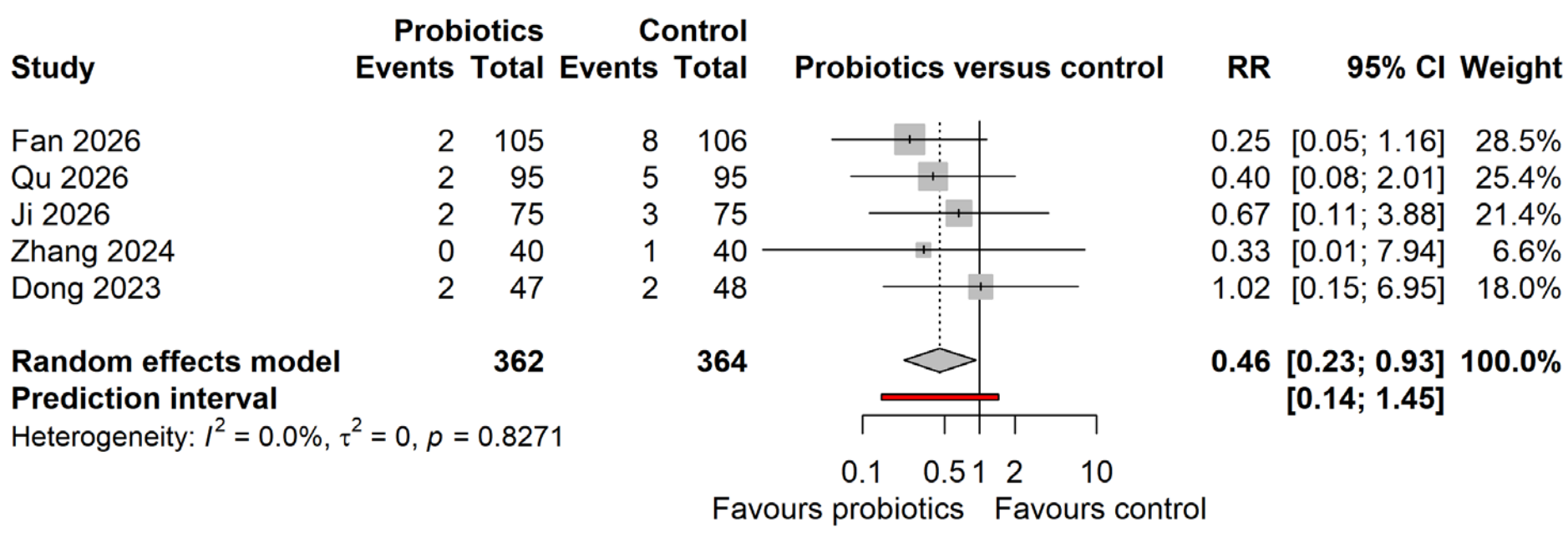

Supplementary Figure S8. Random-effects meta-analysis of nausea.

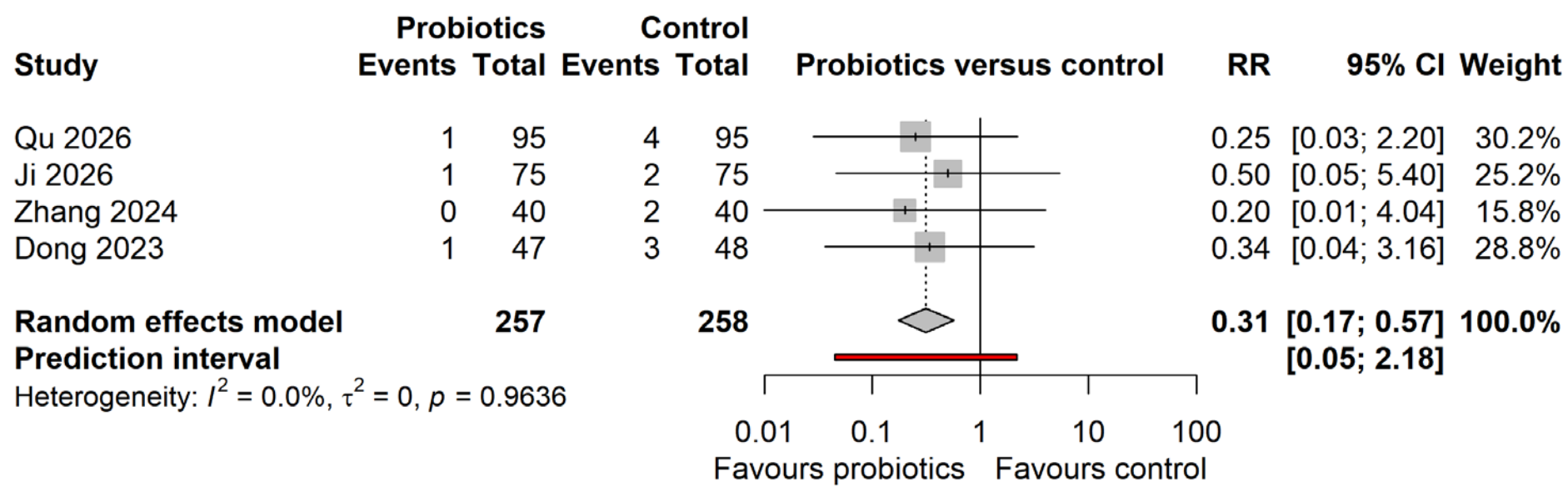

Supplementary Figure S9. Risk of Bias 2 traffic-light plot for overall adverse events.

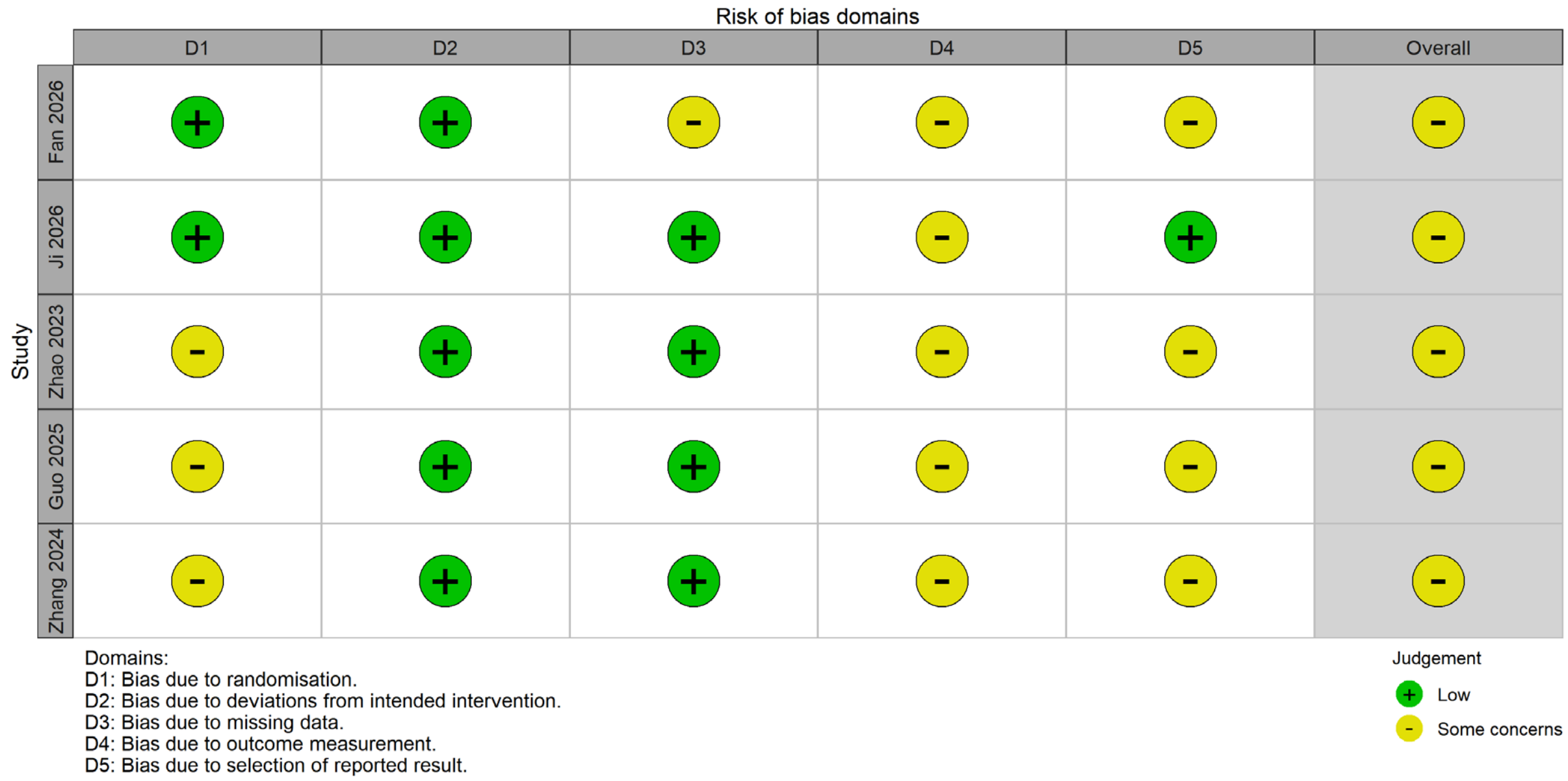

Supplementary Figure S10. Risk of Bias 2 traffic-light plot for intention-to-treat eradication.

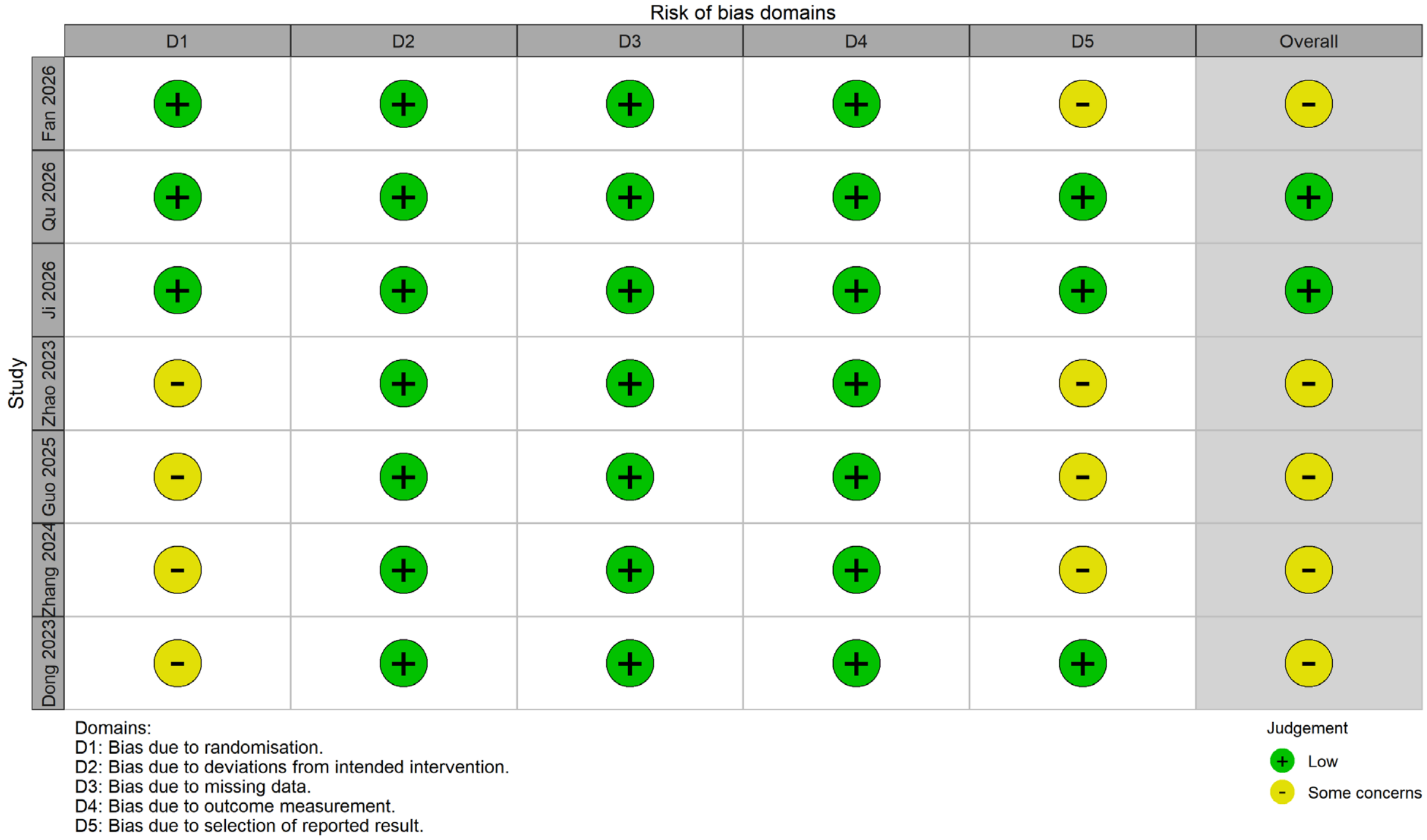

Supplement: Supplementary file 1 [file Supplementaryfile1.pdf]
